# The Loss of Functional Caspase-12 in Europe Is a Pre-Neolithic Event

**DOI:** 10.1371/journal.pone.0037022

**Published:** 2012-05-16

**Authors:** Montserrat Hervella, Theo S. Plantinga, Santos Alonso, Bart Ferwerda, Neskuts Izagirre, Lara Fontecha, Rosa Fregel, Jos W. M. van der Meer, Concepcion de-la-Rúa, Mihai G. Netea

**Affiliations:** 1 Department of Genetics, Physical Anthropology and Animal Physiology, University of the Basque Country, Leioa, Spain; 2 Department of Medicine, Radboud University Nijmegen Medical Centre, Nijmegen, The Netherlands; 3 Department of Genetics, School of Medicine, University of Pennsylvania, Philadelphia, Pennsylvania, United States of America; 4 Department of Genetics, University of La Laguna, La Laguna, Spain; 5 Nijmegen Center for Infection, Inflammation and Immunity (N4i), Nijmegen, The Netherlands; University of Florence, Italy

## Abstract

**Background:**

Caspase-12 (CASP12) modulates the susceptibility to sepsis. In humans, the “C” allele at CASP12 rs497116 has been associated with an increased risk of sepsis. Instead, the derived “T” allele encodes for an inactive caspase-12. Interestingly, Eurasians are practically fixed for the inactive variant, whereas in Sub-Saharan Africa the active variant is still common (∼24%). This marked structure has been explained as a function of the selective advantage that the inactive caspase-12 confers by increasing resistance to infection. As regards to both when positive selection started acting and as to the speed with which fixation was achieved in Eurasia, estimates depend on the method and assumptions used, and can vary substantially. Using experimental evidence, we propose that, least in Eurasia, the increase in the frequency of the T allele might be related to the selective pressure exerted by the increase in zoonotic diseases transmission caused by the interplay between increased human population densities and a closer contact with animals during the Neolithic.

**Methodolog/Principal Findings:**

We genotyped CASP12 rs497116 in prehistoric individuals from 6 archaeological sites from the North of the Iberian Peninsula that date from Late Upper Paleolithic to Late Neolithic. DNA extraction was done from teeth lacking cavities or breakages using standard anti-contamination procedures, including processing of the samples in a positive pressure, ancient DNA-only chamber, quantitation of DNAs by qPCR, duplication, replication, genotyping of associated animals, or cloning of PCR products. Out of 50, 24 prehistoric individuals could finally be genotyped for rs497116. Only the inactive form of CASP12 was found.

**Conclusions/Significance:**

We demonstrate that the loss of caspase-12 in Europe predates animal domestication and that consequently CASP12 loss is unlikely to be related to the impact of zoonotic infections transmitted by livestock.

## Introduction

Caspase-12 (*CASP12*) is a cysteine protease that exerts apoptotic functions, inhibits the inflammatory response and modulates susceptibility to sepsis [1]–[6]. In contrast to most mammals, humans show a nonsense C>T mutation at aminoacid 125 in exon 4 (rs497116), which defines two alleles. The ancestral “C” allele (long L-variant) encodes a full-length functional protein which has been associated with an increased risk of sepsis [7]. The derived “T” allele (short S-variant) encodes for a prematurely terminated, inactive caspase-12. Europeans and Asians are practically fixed for the inactive variant, whereas in Sub-Saharan Africa the active variant is still common (∼24% on average, Table S1). This marked structure has been explained as a function of the selective advantage that the inactive caspase-12 confers by, supposedly, increasing resistance to infection. It is thought that the mutation originated in Africa before the Out-of-Africa expansion of modern humans [8], [9], but as regards to both when positive selection started acting and as to the speed with which fixation was achieved in Eurasia, estimates depend on the method and assumptions used, and can vary substantially. Thus, according to [8], a) the age of the inactive allele can be 980KY if it is considered neutral (no SD provided); b) if instead, the allele is under positive selection (s∼1%), the estimated age is 27,000 years (no SD provided); c) using an alternative phylogenetic method, the age can be 552+/−276 KYA or 397+/−223 KYA or 61+/−16 KYA, depending on the root haplotype considered; d) using a parametric model that predict the spatial pattern of nucleotide diversity and allele-frequency spectrum around the putative target of selection the estimated selection coefficient is 1.7% and the age for the mutation is ∼19KYA (no SD provided); e) using a full-likelihood method then s∼0.8% and the age is ∼29KYA (no SD provided); f) they do not investigate selection from standing variation. On the other hand [9], estimate: a) using the information on non-coding region 4, the age of the allele is between 0 and 154KYA, with an average at 74,250 years ago; b) using a deterministic selection model with parameters *w*_CC_ 0.991, *w*_CT_ 0.999 and *w*_TT_ 1, with h = 0.11, then, the onset of the selective sweep for the T allele would have been between 51–55 KYA; c) under neutrality, the ages of the T allele is estimated as 943KYA; d) They do not investigate selection from standing variation. To this, we have to add the persistence of the active *CASP12* allele in Sub-Saharan Africa.

An assessment of the distribution of allele frequencies of rs497116 in Africa suggests an influence of farming on this locus. Thus, hunter-gatherer populations (Mbuti Pygmies and San) (Table S1) show a T-allele frequency of ∼0.4, whereas for the rest of the populations, farmers, it rises to ∼0.8. The case of the Biaka pygmies, with a frequency of ∼0.8, would seem an exception, but it has been documented that Western Pygmies have had a substantial level of gene flow with neighboring farmers [10]. Therefore, we argue that, at least in Eurasia, the increase in the frequency of the T allele at *CASP12* rs497116 might be related to the selective pressure exerted by husbandry development. Thus, the interplay between increased human population densities and a closer contact with animals, would lead to an increase in zoonotic diseases transmission [11]. In this scenario, the selective advantage of the inactive *CASP12* allele is compatible with the increased resistance to infections that it would confer.

**Figure 1:**
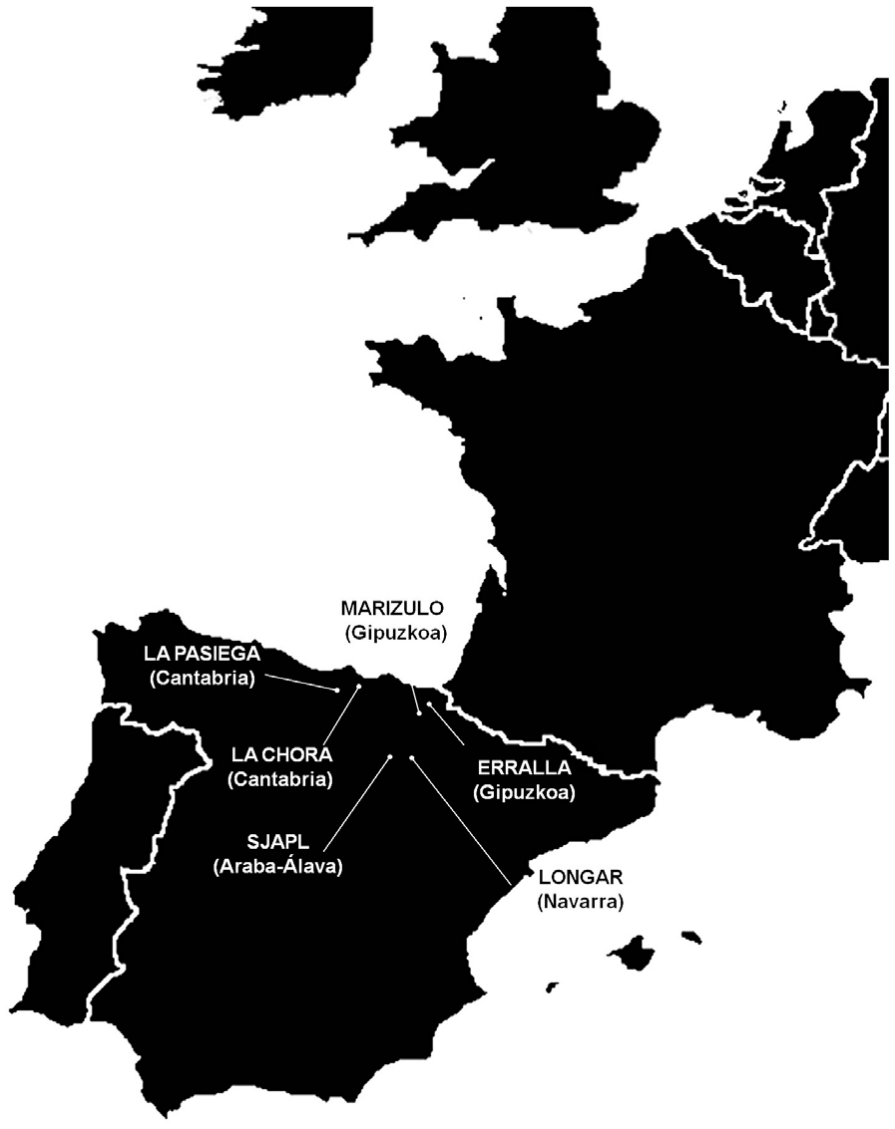
Geographical localization of the ancient human remains analyzed in this work.

We decided to test this hypothesis by genotyping *CASP12* rs497116 in prehistoric individuals from 6 archaeological sites from the North of the Iberian Peninsula (Figure 1) that date from Late Upper Paleolithic to Late Neolithic (Table 1). Faunistic domestic remains suggest that animal husbandry may have started, at least in the Basque Country, ∼6,000-5,500 YBP [12]. Therefore, this sample seems ideal to explore whether the complete loss of caspase-12 function in humans was attained before or after the Neolithic transition. Note that herein we define Neolithic in terms of the development of animal husbandry, regardless of agriculture or other cultural industries. Out of 50, 24 prehistoric individuals could be genotyped for rs497116.

**Table 1:** Temporal and geographical origin of the 24 prehistoric samples analyzed.

Site	N	Cultural Period	^14^C dating (YBP)	ref^b^
La Pasiega (Cantabria)	1	Magdalenian	?	H2012
La Chora (Cantabria)	1	Magdalenian	?	H2012
Erralla (Gipuzkoa)	1	Magdalenian	12,319±190	H2012
Marizulo (Gipuzkoa)	1	Neolithic	5,285±65	H2012
SJAPL^a^ (Araba)	17	Late-Neolithic^c^	5,070±150	IR1999
Longar (Navarre)	3	Neolithic-Calcolithic^c^	4,445±70	IR1999

a SJAPL: San Juan Ante Portam Latinam.

b H2012: see Literature Ref. [26]. IR1999: See Literature Ref. [27].

c based on the associated lithic industry.

## Results and Discussion

We first decided to explore if it is actually possible that Neolithic could have substantially shifted the frequencies of sub-Saharan Africans and Eurasians in a time span ranging from the onset of Neolithic to present time. For Africans, analytical formulae (see Material and methods) show that it is actually possible to increase a mean frequency from 0.4 (frequency before Neolithic; inferred from present-day sub-Saharan African hunter-gatherers) to 0.8 (present-day frequency in sub-Saharan African farmer populations) in ∼200 generations (∼4,000–5,000 years, an estimated time span from the onset of husbandry in sub-Saharan Africa to present time) under a selective regime with a selection coefficient (**s**) of 1%, and a heterozygous effect parameter (**h**) of 0.11, **s** and **h** taken from [8]. Further, our own simulations show that for a neutral allele whose origin is in Africa before the Out-of-Africa expansion and whose frequency in Africa is between 0.3–0.5, the probability that in Europe its frequency at present time is ≥0.95 is 0.021, which rejects the hypothesis of a neutral evolution of *CASP12* rs497116. If we assume that selection on rs497116 is unrelated to animal domestications and that **s** was ∼1% since the origin of the mutation in Africa (before Neolithic, see Material and methods), the probability that in Europe its frequency at present time is ≥0.95 is then 0.024, which also rejects the assumption. We can test instead, what is the expected outcome if selection started in Europe as a consequence of animal domestication. Because the simulation software does not allow us to simulate selection from standing variation we have divided the assessment process in two steps. First, we calculated which would be the expected allele frequency in Europeans at 7,000 years ago under a neutral scenario given that the frequency in Africa is 0.3–0.5 (see Material and Methods). This expected frequency turned out to be ∼0.4. If selection started then in Europe as a consequence of animal domestication, using the same analytical formulae as above (with **s** = 1% and **h** = 0.11, and assuming that population sizes were already big enough), we show that, in Europe, it is possible to go from a frequency of 0.4 to >0.99 in less than 250 generations (5,000–6,000 years). Therefore, if Neolithic exerted such a selective pressure, it can explain present day allele frequencies at *CASP12* rs497116.

Although our simulations use a simple demographic model, they allow us to posit the hypothesis of a Neolithic-driven allele frequency shift scenario with some confidence. Thus, we proceeded with the genotyping stage. Out of 50, 24 prehistoric individuals could be genotyped for rs497116 (by resequencing of a 49 bp region; see Table S2). Only the inactive form of *CASP12* (“T” allele) was found in all samples (Table S1). MtDNA HVR-I resequencing of some of these samples showed: a) that none of the samples was contaminated with DNA from the investigators (Table S3), and b) that despite the lack of diversity at rs497116 there is substantial genetic diversity in this set of samples, confirming the authenticity of our results. Further, sequencing of 10 clones of *CASP12* PCR products from 13 samples, revealed that most clones showed the same haplotype as the original PCR product as expected, although some clones did show some sequence variability outside rs497116 (Table S4), which indicates limited post-mortem damage to the aDNA molecules (rather than contamination with exogenous DNA) [13]. Similarly, replication of the results by independent analysis of a second tooth from a set of 7 individuals in independent laboratories confirmed the authenticity of the results. Finally, the analysis in parallel of *LCT* −13910 T, the SNP responsible for lactase persistence in Europeans, showed a low prevalence (27%) [14] in these Neolithic populations when compared with the current Basque population (66%) [15], which evidences the effect of Neolithic in this trait and adds supports therefore the authenticity of our results.

For the best-represented sample (SJAPL, N = 17) (Table 1) the minimum inactive allele's frequency that is compatible with the observed data is 0.83 (α = 0.05), which suggests that the fixation of the inactive *CASP12* allele in Europe was already achieved before animal domestication, and consequently, before the introduction of an important number of new zoonotic infections. That figure could be pushed further back, i.e. earlier than 10,000 YBP, given the results obtained from the pre-Neolithic samples. It could be argued that the SJAPL site dates to a late Neolithic period, and that therefore there has been plenty of time for domestication to shape the allele frequency distribution of *CASP12*. However, the assignation of SJAPL to late Neolithic is based on the associated lithic industry, as SJAPL is a burial site and not a habitation site. Besides, animal domestication was not yet fully developed at this time, as in habitation sites of this period wild animal remains from hunting activities coexist with faunistic domestic remains in a similar percentage on average [12]. Further, the low prevalence of lactase persistence in our ancient populations (27%) [14] supports that animal exploitation was still a recent event.

In conclusion, we demonstrate that the spread of the inactive allele of *CASP12* in Europe predates animal domestication, and that consequently *CASP12* loss in Eurasians is unlikely to be related to the impact of zoonotic infections transmitted by livestock. It does not look likely either that the ecological change brought about by Neolithic, i.e., anthropogenic modification of the environment affecting the pathogen or pathogen vector's ecology [16], could have triggered the present day distribution of *CASP12* rs497116 alleles. Then, which is the selective agent is responsible for the wide distribution of the *CASP12* rs497116 alleles? Malaria immediately comes to mind, but previous investigations have also ruled out malaria as a selective agent responsible for the persistence of the active *CASP12* allele in Africa [17]. At this point we cannot but speculate that perhaps the predatory/scavenging activity of humans could have triggered a selective response. It would be interesting to know the rs497116 genotypes of earlier humans. Unfortunately, none of the Neanderthal nuclear sequences available overlap this SNP. Perhaps future work analyzing a fair set of early *Homo sapiens* remains could help us bracketing the time point for the expansion of the inactive allele, and thus, we could be able to infer which environmental scenario was responsible for it.

## Materials and Methods

### Estimation of allele frequency after selection using analytical formulae

To assess if the development of agriculture could have driven a shift from a frequency of 0.4 to a present day frequency of 0.8 as a result of selection in Africa we used the following formula [18]:
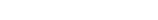
where 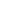 and 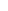 are the allele frequencies of alleles A and a respectively, 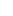 is the frequency of allele A after 1 generation of selection (under constant population size), *w_11_* and *w_12_* are the viabilities of genotypes AA and Aa respectively, and 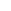 is the mean fitness of the population. If the relative fitness of AA is 1, the relative fitness of Aa will be:
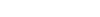
and that of aa, 1-*hs*, where 1-*hs* = *w*_12_/*w*_11_, *h* being the heterozygous effect.

Using this formula as a recursive algorithm, it can be shown that an allele A with an initial frequency of 0.4, under a selective regime with s = 1% and h = 0.11, as suggested by the literature for *CASP12* (see 8) can reach a mean frequency p′ of 0.8 in 200 generations (∼4,000 years).

### Estimation of the probability of allele frequencies by means of simulations

We used SFS_CODE [19], a generalized Wright-Fisher style forward population genetic simulation program for finite-site mutation models with selection, recombination and demography, to infer the probabilities of obtaining an allele frequency greater than or equal to 0.95 in Europe, provided that that allele originated in Africa before the Out-of-Africa expansion of modern humans took place, and that its present day frequency in Africa is between 0.3–0.5 (as observed in African hunter-gatherer populations (Table S1). Note that SFS_CODE does not allow simulating selection from standing variation.

We explored two scenarios. One in which the allele remains neutral along its evolutionary time, and a second one, in which the allele holds a selective coefficient s of 1% (additive) since its origin. The command line, embedded within a Perl script, for the neutral scenario was:

system (“./sfs_code 4 1 -n 100 100 0 100 -L 1 1 –mutation $time_mut -W 0 –additive -t 0 -a N -TS 0.0175 1 3 -TE 0.0175 1 -TE 0.607 -B 0 -b 0 -Td 0 P 0 0.1 -Td 0.004 P 0 10 -Td 0.005 P 0 1.68202 -TS 0.211074 0 1 -Td 0.211074 P 1 0.170737565 -TS 0.534772 1 2 -Td 0.534772 P 1 0.724763218 -Td 0.534772 P 2 0.162342748 -Tg 0.534772 P 1 33.98 -Tg 0.534772 P 2 62.27 -Tm 0.211074 P 0 1 6.133603671 -Tm 0.211074 P 1 0 1.047236559 -Tm 0.534772 P 0 1 0.741012229 -Tm 0.534772 P 0 2 0.472619025 -Tm 0.534772 P 1 0 0.091696045 -Tm 0.534772 P 1 2 0.41215962 -Tm 0.534772 P 2 0 0.013100056 -Tm 0.534772 P 2 1 0.092321359 -Tm 0.54805 P 0 1 0 -Tm 0.54805 P 0 2 0 -Tm 0.54805 P 1 0 0 -Tm 0.54805 P 2 0 0 –trackTrajectory T 0.6 P 0 L 0 S 0 R 0.3 0.5 -o $fich_out -e $fich_err –popFreq $fich_popfreq”);

Note that the program uses a scenario of human evolution based on the demographic parameters used by [20] (excluding their Mexican American population). The parameters are scaled to an effective size of 500 individuals to speed up simulations. Note that in most cases the actual population size does not matter (see SFS_CODE documentation at http://sfscode.sourceforge.net/SFS_CODE/SFS_CODE_home/SFS_CODE_home.html).

We consider 4 simulated populations: present day Africa, Europe at time 7,000 years ago (representing a time point when agriculture started in Europe), present day Asia and present day Europe. Their samples sizes are 100, 100, 0 and 100 respectively. Note also that the variable $time_mut is a random number between 0.001 and 0.003, obtained by a Perl script, indicating the time of origin of the mutation.

The command line for the scenario under additive selection (s = 1%) was:

system (“./sfs_code 4 1 -n 100 100 0 100 -L 1 1 –mutation $time_mut -W 1 10 1 0 –additive -t 0 -a N -TS 0.0175 1 3 -TE 0.0175 1 -TE 0.607 -B 0 -b 0 -Td 0 P 0 0.1 -Td 0.004 P 0 10 -Td 0.005 P 0 1.68202 -TS 0.211074 0 1 -Td 0.211074 P 1 0.170737565 -TS 0.534772 1 2 -Td 0.534772 P 1 0.724763218 -Td 0.534772 P 2 0.162342748 -Tg 0.534772 P 1 33.98 -Tg 0.534772 P 2 62.27 -Tm 0.211074 P 0 1 6.133603671 -Tm 0.211074 P 1 0 1.047236559 -Tm 0.534772 P 0 1 0.741012229 -Tm 0.534772 P 0 2 0.472619025 -Tm 0.534772 P 1 0 0.091696045 -Tm 0.534772 P 1 2 0.41215962 -Tm 0.534772 P 2 0 0.013100056 -Tm 0.534772 P 2 1 0.092321359 -Tm 0.54805 P 0 1 0 -Tm 0.54805 P 0 2 0 -Tm 0.54805 P 1 0 0 -Tm 0.54805 P 2 0 0 –trackTrajectory T 0.6 P 0 L 0 S 0 R 0.3 0.5 -o $fich_out -e $fich_err –popFreq $fich_popfreq”);

### DNA extraction from ancient human remains

DNA extraction was done from teeth lacking cavities or breakages. Anti-contamination procedures [21]–[23] included processing of the samples in a positive pressure, ancient DNA-only chamber (physically separated from the post-PCR laboratory), cleaning of surfaces and material with UV light and sodium hypochlorite, use of disposable gloves, lab coats, caps, shoe covers and masks. To eliminate surface contamination the teeth were washed with a depurinating solution (20% acetic acid, 15% HCl), then with 70% ethanol, and finally rinsed in distilled water. Once dry, the entire tooth surface was irradiated with UV light. The teeth crowns were then cut off with sterile jeweller saws, and the pulp cavity was scraped with sterile dental tools. Each tooth was then incubated in 5 ml of lysis buffer (0.5 M EDTA; 50 mM Tris HCl; SDS 0.5%; 0.01 mg/ml Proteinase K) for 2 h. at 56°C. Subsequently, DNA was extracted by the phenol-chloroform method. Blank tubes were also processed as extraction controls. Extracts were purified with Centricon-30 spin columns (Amicon), and after dilution, three aliquots of 100 µl each were finally stored. For the PCR process, several working dilutions with varying amounts of BSA were tried. All PCRs included negative controls.

### DNA Quantification

We used our standard procedure to quantify the extracted DNA, which consists on measuring the number of molecules of a segment of 113 bp (including primers; 73 bp without primers) of HVR-I of mtDNA by means of qPCR (Step-One, Applied Biosystems). For this, we used oligo 5′-CACCATTAGCACCCAAAGCT-3′ as forward primer and oligo 5′-ACATAGCGGTTGTTGATGGG-3′ as reverse primer. The sequence of the Taqman probe was: VIC-5′-GAAGCAGATTTGGGTAC-3′ (Applied Biosystems).

For each sample four replicates were performed, each in 30 µl containing 1× TaqMan Universal PCR Master Mix (Applied Biosystems), 5 µM each primer, 10 µM probe, and 10 µl DNA extract (diluted 1/10 with BSA). The cycling conditions were 1 cycle of 50°C for 2 min, 95°C for 10 min, followed by 45 cycles of 95°C for 15 s and 60°C for 1 min. in a StepOne Real-Time PCR System.

For the standard curves, serial dilutions of plasmid pCR2.1-new (3.9 Kb) including an insert of 450 bp (Eurofins MWG/Operon) containing the HVR-I region of interest, are included in each experiment to generate standard curves. Two different standard curves were performed. One with 3 points of 1.4*10^4^, 1.4*10^5^ and 1.4*10^6^ molecules/µl for high concentration samples, and a second standard curve for low concentration samples, with 3 points at 1.12*10^4^, 2.24*10^3^ and 448 molecules/µl. Four replicates were used for each dilution point. Typical (%efficiency, r^2^) values were respectively (102%, 0.99) for the high concentration curve, and (83.2%, 0.95) for the low concentration samples. Finally, at least three “no-template-controls” were included with each experiment. Quantification of results can be seen in Table S5.

### CASP12 genotyping

*CASP12* genotyping was done by direct sequencing of PCR products. Each extract was sequenced twice. The PCRs were performed in 25 µl of reaction mixture containing 10 mM Tris-HCL pH 8.3; 2 mM of MgCl2, 0.1 µM of each dNTP, 0.4 µM of each primer, 10 units of AmpliTaq Gold (Applied Biosystems) and 10 µl of diluted DNA (1 µl of DNA extract in 10 µl of 1 mg/ml BSA). Cycling parameter were 96°C 1 min for 1 cycle, followed by 45 cycles of 95°C 15 s, 48°C 30 s, 72° 30 s and a final cycle of 72°C 10 min, using Forward primer 5′-CAACTATCTTCATAATCGAAA-3′ and Reverse primer 5′-TTTTATAACCACTGAGTATCC-3′. These primers define amplicons of 91 bp (49 bp without the primer sequences). Both forward and/or reverse sequences were obtained using the above primers and Rhodamine chemistry in an ABI310 Genetic Analyzer (Applied Biosystems).

### mtDNA HVR I sequencing

The sequencing of the HVR I (15,998–16,400) was carried out by sequencing 6 overlapping fragments of approximately 100 bp each, as described in [24], although in this case the amplification of each fragment was done in independent PCRs. The PCRs were performed in 25 µl of reaction mixture containing 10 mM Tris-HCL pH 8.3; 2 mM of MgCl2, 0.1 µM of each dNTP, 0.4 µM of each primer, 5 units of AmpliTaq Gold (Applied Biosystems) and 10 µl of diluted DNA (1 µl of DNA extract in 10 µl of 1 mg/ml BSA).

Cycling parameter were 95°C for 10 min; followed by 40 cycles of 95°C for 10 sec, annealing temperature for 30 sec, 72°C for 30 sec; and 72°C for 10 min. The annealing temperatures of the primers were as follows: 60°C for the A1/A1R primer pair, 58°C for 2F/2R and 4F/4R, 57°C for 1F/1R and 55°C for 3F/3R and 5F/5R (the primer sequences are listed in [24]. In the event of positive amplification and absence of contamination, the amplifications were purified by Exo SAP IT (USB corporation) and subsequent sequencing in an ABI310 automatic sequencer using chemistry based on dRhodamine. The results obtained were edited with the BioEdit software application (http://www.mbio.ncsu.edu/BioEdit/bioedit.html). Sequences were aligned by eye.

### Reproducibility of the results

a) Duplication of *CASP12* sequencing results

For each of the samples, one extract (one aliquot) was sequenced twice from independent reactions with BigDye 1.1 chemistry using the Reverse primer only. Both sequences were checked to be coincident at rs497116.

b) Cloning of *CASP12* PCR products and sequencing of clones

For 13 samples, *CASP12* PCR products (see above) comprising rs497116 were cloned (TOPO TA Cloning® Kit, Invitrogen) and from each cloning reaction at least 10 colonies were picked up, sequenced and checked for coincident results at rs497116. In this case most clones showed the same haplotype as the original PCR product as expected, although some clones did show some sequence variability outside rs497116. A mean of 1.8 mutations (1.2 A−>G; 0.6 G−>A) per sample cloned (∼80 bp) were found in unique clones, which indicates limited post-mortem damage to the aDNA molecules (rather than contamination with exogenous DNA). (see Table S4).

c) *CASP12* replication in independent laboratories

For the *CASP12* locus, seven samples (five from the SJAPL site and one each from the Erralla and Marizulo sites) were analyzed in duplicate in two different labs (University of the Basque Country and University of La Laguna, Spain). For this, two different teeth from each sample, one for each lab, were extracted and genotyped independently in each lab. Six samples were coincident (TT) and for one sample from SJAPL no PCR product could be obtained in one of the labs.

d) mtDNA sequencing

Because SNP rs497116 is monomorphic in European populations, to increase the chances of detecting possible contamination mitochondrial DNA sequences were obtained from 33 samples by direct sequencing as depicted in Table S5.

e) Duplication of mtDNA sequencing

In addition, for 9 samples the same extract was analyzed twice for their mtDNA (either in the University of the Basque Country, Spain, or in the Radboud University, The Netherlands). MtDNA was chosen because its high variability increases the chances to genetically individualize each sample, and therefore to increase the chances to detect (and track) contamination with exogenous DNA.

f) Replication of mtDNA sequencing

We assessed further the reproducibility of the results obtained in the Univ. of the Basque Country by replicating the analysis of the six overlapping fragments of mtDNA HVRI of a second tooth from each of 2 prehistoric individuals in the University of La Laguna (Spain) (Table S5).

g) Cloning and sequencing of mtDNA PCR products

To further corroborate the results, PCR products corresponding to one of these fragments (∼110 bp) of mtDNA HVRI of 24 different samples (from the La Pasiega, La Chora, Erralla, SJAPL, Longar and Marizulo sites) were cloned (using the TOPO TA Cloning® Kit, Invitrogen). From each cloning reaction at least 10 colonies were picked up, sequenced and checked for coincident results. In this case most clones showed the same haplotype as the original PCR product as expected, although some clones did show some sequence variability at sites outside key mutations. A mean of 5.8 mutations (1.8 A−>G, 2.2G−>A, 1.5 insertion of A, 0.04 insertion of G, 0.2 C−>T; 0.08 T−>C) per sample fragment cloned (∼100 bp) were found in unique clones, possibly as a consequence of post-mortem damage to the aDNA molecules (rather than contamination with exogenous DNA) (see Table S4).

h) Genotyping of individuals involved in the study

Finally, the mtDNA types of most of the personnel that was in contact with the samples was also obtained (see Table S3) in order to minimize the possibility of contamination from the manipulators.

i) Genotyping of animal remains found at the sites

In addition, two dog (*Canis familiaris*) samples, found at the sites the SJAPL and Marizulo respectively, and one chamois (*Rupicapra rupicapra*) from the Erralla site could be analyzed. None of them produced PCR products after amplification with human mtDNA primers or human CASP12 primers, but one dog and the chamois produced the expected sequence identifying the species after amplification with mammalian-specific cytochrome b primers [25]. One dog did not produce positive results with mammalian-specific cytochrome b primers.

## Supporting Information

Table S1 **Distribution of** ***CASP12*** **alleles at aminoacid position 125 (rs497116) in the populations studied and other world populations.** The cytosine (C) from the ancient allele has been substituted in European populations by a thymidine (T), leading to a non-sense mutation and an inactive truncated molecule.
(DOC)

Table S2 **Number of samples analyzed and proportion of which were positive.**
(DOC)

Table S3 **Mitochondrial haplotypes (HVR-I) and** ***CASP12*** **rs497116 genotypes of researchers in this study.**
(DOC)

Table S4 **Mitochondrial DNA and CASP12 sequences of the clones from the samples analyzed in the present study (.xls).**
(XLS)

Table S5 **Summary of quantification, replication and cloning.**
(DOC)
