# Supplementary material for: The Loss of Functional Caspase-12 in Europe Is a Pre-Neolithic Event
Source: PLoS One. 2012 May 16;7(5):e37022. doi: 10.1371/journal.pone.0037022 (PMC3353979; doi:10.1371/journal.pone.0037022)
Supplement: Table S1 — Distribution of CASP12 alleles at aminoacid position 125 (rs497116) in the populations studied and other world populations. The cytosine (C) from the ancient allele has been substituted in European populations by a thymidine (T), leading to a non-sense mutation and an inactive truncated molecule. (DOC) [file pone.0037022.s001.doc]

**Table S1.- Distribution of *CASP12* alleles at aminoacid position 125 (rs497116)** **in the populations studied and other world populations.** The cytosine (C) from the ancient allele has been substituted in European populations by a thymidine (T), leading to a non-sense mutation and an inactive truncated molecule.

|  | **Population^a^** | **2N** | **Dating** | **Caspase 12 allele frequency** |  | **Ref** |
| --- | --- | --- | --- | --- | --- | --- |
|  |  |  |  | **C** | **T** |  |
| **EUROPE** |  |  |  |  |  |  |
|  | Ancient Europeans | 48 | ~4,500-12,500YBP | 0 | 1 | Present Study |
|  | CEU | 120 | contemporary | 0 | 1 | HapMap**^b^** |
|  | ORK | 32 | contemporary | 0 | 1 | Kachapati et al. 2006**^c^** |
|  | BAS | 48 | contemporary | 0 | 1 | Kachapati et al. 2006 |
|  | SAR | 82 | contemporary | 0 | 1 | Kachapati et al. 2006 |
|  | YAK | 50 | contemporary | 0 | 1 | Kachapati et al. 2006 |
|  | RUS | 78 | contemporary | 0 | 1 | Kachapati et al. 2006 |
|  | FRE | 58 | contemporary | 0 | 1 | HGDP-CEPH |
|  | TUS | 16 | contemporary | 0 | 1 | HGDP-CEPH |
|  | ITA-N | 28 | contemporary | 0 | 1 | HGDP-CEPH |
|  | ADY | 34 | contemporary | 0 | 1 | HGDP-CEPH |
| **North Africa** |  |  |  |  |  |  |
|  | MOZ | 60 | contemporary | 0.0165 | 0.983 | Kachapati et al. 2006 |
| **Sub-Saharan Africa** |  |  |  |  |  |  |
|  | ASW | 106 | contemporary | 0.113 | 0.887 | HapMap |
|  | YRI | 226 | contemporary | 0.142 | 0.858 | HapMap |
|  | YRI-B | 54 | contemporary | 0.1665 | 0.8335 | Kachapati et al. 2006 |
|  | LWK | 180 | contemporary | 0.211 | 0.789 | HapMap |
|  | MKK | 286 | contemporary | 0.077 | 0.923 | HapMap |
|  | MAN | 50 | contemporary | 0.220 | 0.780 | Kachapati et al. 2006 |
|  | PYG-B | 72 | contemporary | 0.2505 | 0.7505 | Kachapati et al. 2006 |
|  | PYG-M | 28 | contemporary | 0.607 | 0.393 | Kachapati et al. 2006 |
|  | BAN-A | 28 | contemporary | 0.0355 | 0.9645 | Kachapati et al. 2006 |
|  | BAN-B | 18 | contemporary | 0.2775 | 0.7225 | Kachapati et al. 2006 |
|  | SAN | 12 | contemporary | 0.583 | 0.417 | Kachapati et al. 2006 |
|  | MAL | 194 | contemporary | 0.220 | 0.780 | Ferwerda et al. 2009 |
| MIDDLE-EAST |  |  |  |  |  |  |
|  | BED | 98 | contemporary | 0.0205 | 0.9795 | Kachapati et al. 2006 |
|  | DRU | 96 | contemporary | 0.0105 | 0.9895 | Kachapati et al. 2006 |
|  | PAL | 102 | contemporary | 0.049 | 0.951 | Kachapati et al. 2006 |
| East-Asia |  |  |  |  |  |  |
|  | CHB | 168 | contemporary | 0.006 | 0.994 | HapMap |
|  | CHB-B | 358 | contemporary | 0.0085 | 0.9915 | Kachapati et al. 2006 |
|  | JPT | 88 | contemporary | 0 | 1 | HapMap |
|  | JPT-B | 60 | contemporary | 0 | 1 | Kachapati et al. 2006 |
|  | MEL | 26 | contemporary | 0 | 1 | Kachapati et al. 2006 |
|  | CAM | 16 | contemporary | 0 | 1 | Kachapati et al. 2006 |
|  | PAP | 20 | contemporary | 0 | 1 | Kachapati et al. 2006 |
| SOUTH-ASIA |  |  |  |  |  |  |
|  | BAL | 48 | contemporary | 0.0625 | 0.9375 | HapMap |
|  | BRA | 50 | contemporary | 0 | 1 | HapMap |
|  | BUR | 50 | contemporary | 0.02 | .98 | Kachapati et al. 2006 |
|  | HAZ | 50 | contemporary | 0 | 1 | Kachapati et al. 2006 |
|  | KAL | 50 | contemporary | 0 | 1 | Kachapati et al. 2006 |
|  | MAK | 48 | contemporary | 0.021 | 0.979 | Kachapati et al. 2006 |
|  | PAT | 50 | contemporary | 0 | 1 | Kachapati et al. 2006 |
|  | SIN | 50 | contemporary | 0.02 | 0.98 | Kachapati et al. 2006 |
| AMERICAS |  |  |  |  |  |  |
|  | GIH | 176 | contemporary | 0.034 | 0.966 | HapMap |
|  | PIM | 92 | contemporary | 0 | 1 | Kachapati et al. 2006 |
|  | KAR | 78 | contemporary | 0 | 1 | Kachapati et al. 2006 |
|  | COL | 26 | contemporary | 0 | 1 | HGDP-CEPH |

^a^Population descriptors

ADY: Adygei from Caucasus; ASW: African ancestry in Southwest USA; BAL: Balochi from Pakistan; BAN-A: Bantu N. E. Kenya; BAN-B: Bantu from South Africa; BAS: French Basque from France; BED: Bedouin from Israel; BRA: Brahui from Pakistan; BUR: Burusho from Pakistan; CAM: Cambodians from Cambodia; CEU: Utah residents with Northern and Western European ancestry from the CEPH collection; CHB: Han Chinese in Beijing, China; CHB-B: Han, Tujia, Yizu, Dai, Lahu, Miaozu, She, Orogen, Naxi, Daur, Tu, Mongola, Hezhen, Xibo, and Uygur populations from China; DRU: Druze from Israel; COL: Colmbians from Colombia; FRE: French from France; GIH: Gujarati Indians in Houston, TexasÇ; HAZ: Hazara from Pakistan; ITA-N: North Italians from Bergamo, Italy; JPT: Japanese in Tokyo, Japan; JPT-B: Japanese from Japan; KAL: Kalash from Pakistan; KAR: Kartitiana, Suruir from Brazil; LWK: Luhya in Webuye, Kenya; MAK: Makrani from Pakistan; MAN: Mandenka from Senegal; MEL: Melanesians from Bougainville; MKK: Maasai in Kinyawa, Kenya; MOZ: Mozabite from Algeria; ORK: Orcadians from Scotland; PAL: Palestinians from Israel; PAP: Papuans from New Guinea; PAT: Pathan from Pakistan; PIM: Pima, Maya from Mexico; PYG-B: Biaka Pymies from Central Africa; PYG-M: Mbuti Pymies from Congo; RUS: Russians from Russia.; SAN: San from Namibia; SAR: Sardinians form Italy; SIN: Sindhi from Pakistan; TUS: Tuscans from Italy; YAK: Yakut from Russia; YRI: Yoruban in Ibadan, Nigeria; YRI-B: Yoruba from Nigeria;

^b^HapMap: The Internation al HapMap project:( http://hapmap.ncbi.nlm.nih.gov/cgi-perl/gbrowse/hapmap28_B36/); [HGDP - CEPH](http://www.cephb.fr/en/hgdp/main.php)**:** Human Genome Diversity Genotype Database V3.0 (**http://www.cephb.fr/en/hgdp/main.php**).

^c^Kachapati K, O'Brien TR, Bergeron J, Zhang M, Dean M (2006). Population distribution of the functional caspase-12 allele. Hum Mutat 27: 975.
