# Supplementary material for: The Loss of Functional Caspase-12 in Europe Is a Pre-Neolithic Event
Source: PLoS One. 2012 May 16;7(5):e37022. doi: 10.1371/journal.pone.0037022 (PMC3353979; doi:10.1371/journal.pone.0037022)
Supplement: Table S2 — Number of samples analyzed and proportion of which were positive. (DOC) [file pone.0037022.s002.doc]

**Table S2.- Number of samples analyzed and proportion of which were positive**

| **Site** | **Initial number of samples** | **positive for casp-12^a^** | **positive for mtDNA ^a^** |
| --- | --- | --- | --- |
| SJAPL | 26 | 17/26 | 21/26 |
| Longar | 20 | 3/20 | 8/20 |
| Marizulo | 1 | 1/1 | 1/1 |
| La Pasiega | 1 | 1/1 | 1/1 |
| La Chora | 1 | 1/1 | 1/1 |
| Erralla | 1 | 1/1 | 1/1 |
| TOTAL | 50 | 24/50 | 33/50 |

^a^Positive means that identical sequence obtained in 2 aliquots of the same extract.

^b^Not all available samples were analyzed for their mtDNA haplotype.
