# Supplementary material for: The Loss of Functional Caspase-12 in Europe Is a Pre-Neolithic Event
Source: PLoS One. 2012 May 16;7(5):e37022. doi: 10.1371/journal.pone.0037022 (PMC3353979; doi:10.1371/journal.pone.0037022)
Supplement: Table S3 — Mitochondrial haplotypes (HVR-I) and CASP12 rs497116 genotypes of researchers in this study. (DOC) [file pone.0037022.s003.doc]

**Table S3.- Mitochondrial haplotypes (HVR-I) and *CASP12* rs497116 genotypes of researchers in** this study

| **Lab** | **researcher** | **HVR-I haplotype^a^** | ***CASP12* rs497116** |
| --- | --- | --- | --- |
| Univ. Basque Country (Spain) | Researcher #1 | CRS | T/T |
| Univ. Basque Country (Spain) | Researcher #2 | CRS | T/T |
| Univ. Basque Country (Spain) | Researcher #3 | 189 | T/T |
| Univ. Basque Country (Spain) | Researcher #4 | 319 | T/T |
| Univ. Basque Country (Spain) | Researcher #5 | 092-224-311 | T/T |
| Univ. La Laguna (Spain) | Researcher #1 | 129-316 | NA^b^ |
| Univ. La Laguna (Spain) | Researcher #2 | 223-278-311-362 | T/T |
| Radboud Univ. (The Netherlands) | Researcher #1 | CRS | T/T |
| Archaeologists | Researcher #1 | 304 | T/T |
| Archaeologists | Researcher #2 | 291 | T/T |
| Archaeologists | Researcher #3 | 220 | T/T |
| Archaeologists | Researcher #4 | 051-162-264 | T/T |
| Archaeologists | Researcher #5 | 298 | T/T |

^a^HVR-I: Hypervariable Region I of mtDNA. CRS: Cambridge Reference Sequence.The figures correspond to the position in region I of HVR of mt DNA that change with respect to the CRS. Precise mitochondrial coordinates can be obtained by adding 16,000.

^b^ NA not analyzed
