# Supplementary material for: The Loss of Functional Caspase-12 in Europe Is a Pre-Neolithic Event
Source: PLoS One. 2012 May 16;7(5):e37022. doi: 10.1371/journal.pone.0037022 (PMC3353979; doi:10.1371/journal.pone.0037022)
Supplement: Table S5 — Summary of quantification, replication and cloning. (DOC) [file pone.0037022.s005.doc]

**Table S5**.- Summary of quantification, replication and cloning

| **Sample^a^** | **Site** | **Quantification molec/μl^b^** | **HVR-I haplotype ^c, d, e^** | **Caspase-12 PCR^f^** | **Replication by lab^g^** | **clones sequenced^h^** |
| --- | --- | --- | --- | --- | --- | --- |
| CH | CHORA | 669.20 | 093-362 | + | NA | Caspase-12^k^; mtDNA^i^ |
| ERR | ERRALLA | 667.00 | 270 | + | (CASP12) La Laguna (mtDNA) La Laguna | mtDNA^i^ |
| LO-1 | LONGAR | 0.00 | PCR FAILED | NA | NA | NA |
| LO-2 | LONGAR | 0.00 | PCR FAILED | NA | NA | NA |
| LO-3 | LONGAR | 367.4 | 176 | NA | NA | NA |
| LO-4 | LONGAR | 0.00 | PCR FAILED | NA | NA | NA |
| LO-5 | LONGAR | 0.00 | PCR FAILED | NA | NA | NA |
| LO-6 | LONGAR | 0.00 | PCR FAILED | NA | NA | NA |
| LO-7 | LONGAR | 543.3 | 320 | NA | NA | NA |
| LO-8 | LONGAR | 287 | 319 | NA | NA | NA |
| LO-9 | LONGAR | 0.00 | PCR FAILED | NA | NA | NA |
| LO-10 | LONGAR | 582.10 | 319 | + | NA | Caspase-12^k^; mtDNA^i^ |
| LO-11 | LONGAR | 1,164.00 | 270-319 | + | NA | mtDNA^i^ |
| LO-12 | LONGAR | 0.00 | PCR FAILED | NA | NA | NA |
| LO-13 | LONGAR | 0.00 | PCR FAILED | NA | NA | NA |
| LO-14 | LONGAR | 292.55 | 189 | NA | NA | NA |
| LO-15 | LONGAR | 0.00 | PCR FAILED | NA | NA | NA |
| LO-16 | LONGAR | 0.00 | PCR FAILED | NA | NA | NA |
| LO-17 | LONGAR | 387.11 | 266-268-270-311 | NA | NA | NA |
| LO-18 | LONGAR | 0.00 | PCR FAILED | NA | NA | NA |
| LO-19 | LONGAR | 0.00 | PCR FAILED | NA | NA | NA |
| LO-20 | LONGAR | 2,829.00 | 69-126-278-311 | + | NA | Caspase-12^k^; mtDNA^i^ |
| MZ | MARIZULO | 766.70 | 270 | + | (CASP12) La Laguna (mtDNA) La Laguna | mtDNA^i^ |
| PS | PASIEGA | 1,229.60 | CRS | + | NA | Caspase-12^k^; mtDNA^i^ |
| SJ-23 | SJAPL | 536.23 | 069-126 | + | (CASP12) La Laguna | mtDNA^i^ |
| SJ-65 | SJAPL | 1,771.50 | CRS | + | NA | mtDNA^i^ |
| SJ-66 | SJAPL | 0.00 | PCR FAILED | NA | NA | NA |
| SJ-67 | SJAPL | 1,155.30 | 126-294 | + | NA | mtDNA^i^ |
| SJ-68 | SJAPL | 275.11 | 069-126 | NA | NA | NA |
| SJ-69 | SJAPL | 2,414.40 | CRS | + | NA | Caspase-12^k^; mtDNA^i^ |
| SJ-70 | SJAPL | 686.20 | 51 | + | NA | Caspase-12^k^; mtDNA^i^ |
| SJ-71 | SJAPL | 0.00 | PCR FAILED | NA | NA | NA |
| SJ-72 | SJAPL | 1,691.00 | CRS | + | NA | Caspase-12^k^; mtDNA^i^ |
| SJ-73 | SJAPL | 1,070.50 | CRS | + | NA | Caspase-12^k^; mtDNA^i^ |
| SJ-74 | SJAPL | 1,758.50 | 93 | + | NA | Caspase-12^k^; mtDNA^i^ |
| SJ-75 | SJAPL | 2,624.70 | 311 | + | NA | mtDNA^i^ |
| SJ-76 | SJAPL | 6,759.30 | 221-224-257-259-261-270-296 | + | NA | mtDNA^i^ |
| SJ-77 | SJAPL | 2777.31 | CRS | PCR FAILED | NA | NA |
| SJ-78 | SJAPL | 15,169.30 | 129 | + | NA | Caspase-12^k^; mtDNA^i^ |
| SJ-79 | SJAPL | 1,365.40 | 129-224-362-376 | + | NA | Caspase-12^k^, mtDNAi |
| SJ-80 | SJAPL | 0.00 | PCR FAILED | NA | NA | NA |
| SJ-81 | SJAPL | 273.10 | CRS | NA | NA | NA |
| SJ-82 | SJAPL | 2,586.30 | 126-194 | + | NA | Caspase-12^k^; mtDNA^i^ |
| SJ-83 | SJAPL | 0.00 | PCR FAILED | NA | NA | NA |
| SJ-84 | SJAPL | 3,612.40 | 093-224-311-360 | + | NA | Caspase-12^k^; mtDNA^i^ |
| SJ-105 | SJAPL | 149.21 | CRS | + | (CASP12) La Laguna | mtDNA^i^ |
| SJ-234 | SJAPL | 7,348.00 | 224-311 | + | (CASP12) La Laguna | mtDNA^i^ |
| SJ-242 | SJAPL | 9,238.00 | 069-126-278 | + | (CASP12) La Laguna | mtDNA^i^ |
| SJ-246 | SJAPL | 30,344.00 | 270 | PCR FAILED | PCR FAILED | NA |
| SJ-266 | SJAPL | 1,241.00 | 311 | PCR FAILED | NA | NA |

^a^ total number of samples considered here: 50 (24 *CASP12* positive samples, 9 positive for mtDNA). Positive means the same genotype/haplotype obtained twice in two different aliquots of the same extract (same tooth).

^b^ Estimated number of mtDNA molecules per microlitre in a 1/10 working dilution (including BSA) of the samples

^c^ HVR-I: Hypervariable Region I of mtDNA

^d^ CRS: Cambridge Reference Sequence.

^e^ The figures correspond to the position in region I of HVR of mtDNA that change with respect to the CRS. Precise mitochondrial coordinates can be obtained by adding 16,000.

^f^ NA: Not Attempted

^g^ NA: Not Attempted

^h^ NA: Not Attempted

^i^ 1 fragment, 10 sequences

^j^ 11 sequences with Reverse primer plus 4 sequences with Forward primer

^k^ 10 sequences with Reverse primer

^l^ 10 sequences with Reverse primer plus 4 sequences with Forward primer
